# Transcranial photobiomodulation enhances visual working memory capacity in humans

**DOI:** 10.1126/sciadv.abq3211

**Published:** 2022-12-02

**Authors:** Chenguang Zhao, Dongwei Li, Yuanjun Kong, Hongyu Liu, Yiqing Hu, Haijing Niu, Ole Jensen, Xiaoli Li, Hanli Liu, Yan Song

**Affiliations:** ^1^ State Key Laboratory of Cognitive Neuroscience and Learning & IDG/McGovern Institute for Brain Research, Beijing Normal University, Beijing, China.; ^2^ Center for Cognition and Neuroergonomics, State Key Laboratory of Cognitive Neuroscience and Learning, Beijing Normal University at Zhuhai, Guangdong, China.; ^3^ School of Systems Science, Beijing Normal University, Beijing, China.; ^4^ Centre for Human Brain Health, School of Psychology, University of Birmingham, Birmingham, UK.; ^5^ Department of Bioengineering, University of Texas at Arlington, Arlington, TX, USA.

## Abstract

Transcranial photobiomodulation (tPBM) is a safe and noninvasive intervention that has shown promise for improving cognitive performance. Whether tPBM can modulate brain activity and thereby enhance working memory (WM) capacity in humans remains unclear. In this study, we found that 1064-nm tPBM applied to the right prefrontal cortex (PFC) improves visual working memory capacity and increases occipitoparietal contralateral delay activity (CDA). The CDA set-size effect during retention mediated the effect between the 1064-nm tPBM and subsequent WM capacity. The behavioral benefits and the corresponding changes in the CDA set-size effect were absent with tPBM at a wavelength of 852 nm or with stimulation of the left PFC. Our findings provide converging evidence that 1064-nm tPBM applied to the right PFC can improve WM capacity.

## Teaser

Transcranial photobiomodulation (at 1064 nm) applied to the right prefrontal cortex improves visual working memory capacity.

## INTRODUCTION

Working memory (WM), the ability to actively store useful information “in mind” over seconds, plays a vital role in many cognitive functions. Individual differences in WM capacity predict fluid intelligence and broad cognitive function (*1*), which has made increasing WM capacity become an attractive aim for interventions and enhancement. In the past decades, noninvasive brain stimulation (NIBS) technology involving transcranial application of electrical (direct or alternating) or magnetic fields to the specific scalp or multiple brain circuits has been proven to be useful for improving WM performance. Research using NIBS has found that behavioral enhancement is associated with neurophysiological changes, including increased interregional functional connectivity (*2*) and oscillatory neuronal activity (*3*) as well as changes in event-related potentials (ERPs) (*4*).

Transcranial photobiomodulation (tPBM) is a noninvasive light illumination method that targets the brain at wavelengths between 600 and 1100 nm. Recently, tPBM has been applied to modulate metabolic processes in the brain, and it has emerged as a promising intervention to improve cognitive functions. It has been suggested that tPBM up-regulates complex IV of the mitochondrial respiratory chain to modulate cytochrome c oxidase (CCO). This leads to increased adenosine triphosphate (ATP) formation and initiates secondary cell signaling pathways (*5*–*7*). The resulting metabolic effects following PBM increase cerebral metabolic energy production, oxygen consumption, and blood flow in animals and humans (*8*–*11*). In addition, several studies suggest that PBM can (i) enhance neuroprotection by modulating neurotrophic factors and inflammatory signaling molecules as well as anti-apoptotic mediators (*12*), (ii) activate ion channels (*13*), and (iii) stimulate transcription factors that up-regulate the expression levels of genes (*14*). In an Alzheimer’s disease (AD) mouse model, tPBM can reduce perivascular microglia and promote angiogenesis to further enhance Aβ clearance (*15*). In particular, in the past 2 years, several clinical studies have provided convincing evidence that tPBM improves cognition in patients with AD and dementia (*16*, *17*) and facilitates treatment for other neurological disorders (*18*, *19*).

Regarding WM, Rojas *et al.* (*20*) demonstrated that tPBM could improve prefrontal cortex (PFC) oxygen consumption and metabolic energy, thereby increasing PFC-based memory functions in rats. Other studies have shown that 1072-nm tPBM can reverse WM deficits in middle-aged mice (*21*). These animal findings suggest that the oxygen metabolism of cortical tissue exposed to tPBM is enhanced and that this can explain the improved memory. Two human behavioral studies have shown that 1064-nm tPBM over the right PFC can improve accuracy and speed up reaction time in WM tasks (*22*, *23*). Meanwhile, other behavioral studies suggested that high-order cognitive functions, such as sustained attention and emotion (*22*), as well as executive functions (*24*), could also be improved after tPBM therapy.

**Fig. 1.**
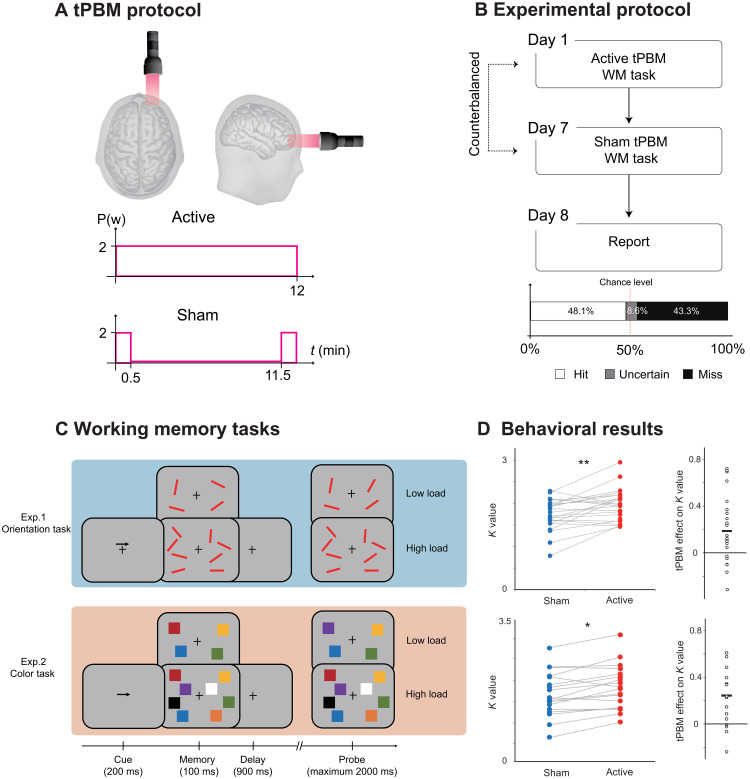
Protocol, task, and behavioral results in experiments 1 and 2. (**A**) tPBM protocol. Active tPBM was delivered by a laser with 1064 nm to the right PFC for a total of 12 min. (**B**) Experimental protocol. Each participant received two tPBM sessions (active and sham, randomized, and double-blinded design) separated by 1 week. On the eighth day, participants were required to report or guess which session involved active or sham tPBM. (**C**) WM tasks. In experiment 1, the participants were required to perform an orientation WM task. In experiment 2, the participant was required to perform a color WM task. Two tasks used the same relative timing and protocol, and the only difference between the two tasks was the memory dimension (orientation in experiment 1 and color in experiment 2). Each participant only participated in one experiment. (**D**) Left: Performance in terms of *K* values for orientation WM task (up) and color WM task (down) under sham tPBM (blue circles) and active tPBM (red circles). Right: The tPBM effect on the *K* values (active minus sham). The dots indicate individual performance. ^*^P < 0.05 and **P < 0.01.

However, even the simplest WM task involves multiple cognitive processes, such as perceptual encoding, selective attention, and motor execution, which might confound the associations between the tPBM effect and WM enhancement. Taking this into account, we chose the *K* values estimates to assess the accurate number of items maintained in the visual WM for the given load array (*25*). Given that the right PFC is associated with information maintenance in WM (*26*), we hypothesized that 1064-nm tPBM over the right PFC (Fig. 1A) leads to enhancements in visual WM capacity. However, we still lack WM-related neurological evidence to directly bridge the gap between tPBM effects and WM behavioral benefits. Previous studies have extensively demonstrated that contralateral delay activity (CDA) tracks the number of objects stored in visual WM. Furthermore, the set-size effects of the CDA (defined as the increase in amplitudes from set-size two to set-size four) predicted the individual differences in WM capacity (*27*). Thus, we linked behavioral benefits (*K* values) in WM capacity from tPBM with ERP biomarkers (CDA) of WM capacity. As the research on tPBM as a potential tool for effective NIBS is in its early stages, several key questions exist. For example, a good understanding of tPBM-evoked electrophysiological effects in the human brain is lacking.

We conducted four double-blind, sham-controlled tPBM experiments (Fig. 1A), in which participants completed two different sessions of tPBM separated by a week, with sham or active tPBM on the PFC (Fig. 1B). After stimulation, participants performed a classical change detection task in which WM load was manipulated (high versus low load; Fig. 1C), while the electroencephalography (EEG) was recorded. The classical change detection task required participants to maintain the features of items (orientation for experiment 1; color for experiment 2) at the cued side in WM for subsequent recognition, which reliably induces sustained CDA components. Then, we performed a series of follow-up experiments that explored the specificity of tPBM in terms of wavelengths (experiment 3) and stimulation site (experiment 4) on the enhancement of WM capacity.

## RESULTS

### 1064-nm tPBM on the right PFC enhanced individual WM capacity

Two classic change detection tasks were implemented to assess WM performance, which required the participants to remember the orientations (experiment 1) or color (experiment 2) of a set of items in the cued hemifield (see Fig. 1C). We calculated the visual memory capacity using a standard formula (*27*) that essentially assumes that if an observer can hold in memory *K* items from an array of *S* items, then the item that changed should be one of the items being held in memory on *K*/*S* trials, leading to correct performance on *K*/*S* of the trials in which an item changed. The formula for memory capacity is *K* = *S* × (*H* − *F*), where *S* is the size of the presented array, *H* is the observed hit rate, and *F* is the false alarm rate. We evaluated the WM capacity according to the *K* values under the high-load condition. A two-way mixed-effect analysis of variance (ANOVA) with tPBM stimulation (active and sham; within-subjects) and tasks (orientation and color; between-subjects) as factors was conducted with *K* values as the dependent variable. The results revealed a significant main effect of tPBM stimulation (*F*_1,40_ = 13.436, *P* < 0.001, η*_P_^2^* = 0.925) but no significant interaction between tPBM stimulation and task (*F*_1,40_ = 0.080, *P* = 0.779, η*_P_^2^* = 0.006). Specifically, compared with sham tPBM, the *K* values increased after 1064-nm tPBM both in the orientation WM task (experiment 1: *t*_22_ = 2.841 and *P* = 0.009, two-tailed) and in the color WM task (experiment 2: *t*_17_ = 2.760 and *P* = 0.013, two-tailed). The mean tPBM effect (active minus sham) on the *K* values for experiment 1 was 0.186 ± 0.065 (BF_10_ = 5.212 and Cohen’s *d* = 0.568), and the mean tPBM effect on *K* values for experiment 2 was 0.188 ± 0.051 (BF_10_ = 20.336 and Cohen’s *d* = 0.651). These results support the hypothesis that 1064-nm tPBM on the right PFC enhances WM capacity.

Several studies investigating neuro-enhancement using transcranial direct current stimulation (tDCS) have shown improved WM with stimulation but mainly for individuals with low WM capacity (*4*, *28*). To examine this effect with respect to tPBM, we divided participants into two subgroups based on their *K* values in the orientation WM task during sham tPBM stimulation in experiment 1 (*n* = 11 and *n* = 12 for low- and high-performance groups, respectively). A two-way mixed-effects ANOVA showed no significant interaction between tPBM stimulation and subgroup (*F*_1,22_ = 1.170, *P* = 0.291, η*_P_^2^* = 0.110). Therefore, participants with both good and poor WM capacity improved after 1064-nm tPBM. A similar analysis involving the color WM task also showed no performance-dependent effects in experiment 2 (*F*_1,17_ = 0.002, *P* = 0.963, η*_P_*^2^ < 0.001).

Our results in experiments 1 and 2 demonstrate that participants could maintain more items in visual WM with external 1064-nm tPBM stimulation on the right PFC. These effects were independent of prior WM ability for both orientation and color. Participants could not report or guess whether they were assigned to the sham or active tPBM group. Subjects guessed at chance level (see Fig. 1B, hit rate = 48.1%), suggesting that they had no awareness of whether they received active tPBM.

### CDA tracks the enhancement in individual WM capacity

**Fig. 2.**
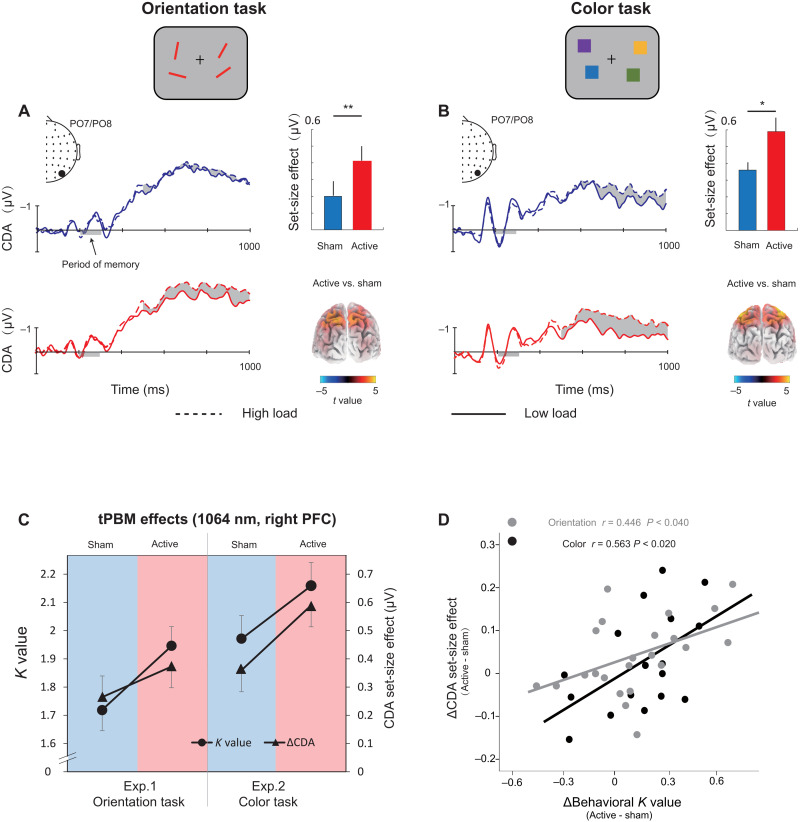
Grand average of ERPs and its link with behavior. (**A**) The orientation WM task in experiment 1 and (**B**) the color WM task in experiment 2. Shading indicates the CDA set-size effect. The enlarged black dots on EEG topographies show PO7/PO8 electrodes. Bar plots represent the average CDA set-size effect. Error bars represent SEM. Significant set-size effects are located in the IPS. Three-dimensional (3D) brain map (t-map; posterior view) of significant tPBM effect on CDA. (**C**) tPBM-effect. *K* values and CDA set-size effect for the two tasks (orientation WM task and color WM task) and two sessions (active tPBM and sham tPBM). (**D**) Scatterplots of participants’ behavioral benefits (active minus sham) against the changes in the CDA set-size effect (active minus sham) for the orientation WM task (gray) and the color WM task (black). *P < 0.05 and **P < 0.01.

The EEG signals were recorded while participants performed the WM tasks. Consistent with previous studies (*27*), the ERP results show a negative deflection at contralateral relative to ipsilateral scalp sites at PO7 and PO8 (see the Supplementary Materials). We defined the CDA set-size effect as the CDA amplitude with respect to the maintenance of *S* = 2 objects (low load) minus the amplitude of maintenance of *S* = 4 objects (high load). Note that we did not track the “raw” CDA amplitude but rather used the increase in CDA amplitude from low to high WM load as the dependent variable. To investigate the effect of tPBM on the CDA set-size effect, a two-way mixed ANOVA on the CDA set-size effect was conducted considering the WM task (orientation and color) and tPBM stimulation (active and sham) as factors. As expected, the results showed a significant main effect of tPBM stimulation (*F*_1,40_ = 12.249, *P* = 0.001, η*_P_*^2^ = 0.227). The main effect of task (*F*_1,40_ = 0.660, *P* = 0.421, η*_P_*^2^ = 0.012) and the tPBM stimulation × task interaction (*F*_1,40_ = 0.474, *P* = 0.495, η*_P_*^2^ = 0.011) was not significant. Follow-up *t* tests indicated that the CDA set-size effect during the delay period was significantly stronger in the active tPBM session than in the sham tPBM session for both the orientation WM task (*t*_22_ = 2.313, *P* = 0.030, Cohen’s *d* = 0.463, two-tailed) and color WM task (*t*_17_ = 2.506, *P* = 0.023, Cohen’s *d* = 0.591, two-tailed). Source estimates using standardized low resolution electromagnetic tomography (sLORETA; see Materials and Methods) of the CDA set-size effect are shown in Fig. 2. These results demonstrated that the increase in CDA set-size effects (active minus sham) was localized in the superior intraparietal sulcus (IPS) for two WM tasks with 1064-nm tPBM applied over the right PFC (*P*s < 0.05).

To better understand the tPBM effect, we show the *K* values and CDA set-size effects for the different stimulation sessions with respect to the orientation and color WM tasks (Fig. 2C). Although the *K* values and CDA set-size effects were evaluated as separate dimensions involving WM, as can be seen here, the changes in *K* values and CDA across the two tasks have similar trends as when compared active tPBM to the sham tPBM. These results are consistent with the hypothesis that both WM improvement and CDA increases are associated with the tPBM effect. As expected, the CDA set-size effect from the two experiments shows a task-independent tPBM effect.

Next, we tested whether the EEG data provided evidence of the beneficial tPBM effect. We performed Pearson correlation analyses at the subject level to provide more detailed information on the relationships between CDA and behavior. As shown in Fig. 2D, the differences in CDA set-size effects between active and sham sessions were correlated positively with the behavioral differences between these sessions (for orientation task: *r* = 0.446 and *P* < 0.040; for color task: *r* = 0.563 and *P* < 0.020). The results suggest that, for both color and orientation WM tasks, the tPBM-associated changes in CDA set-size effects can predict tPBM-associated behavioral benefits.

### CDA mediates the WM improvement with tPBM (1064 nm) applied to the right PFC

**Fig. 3.**
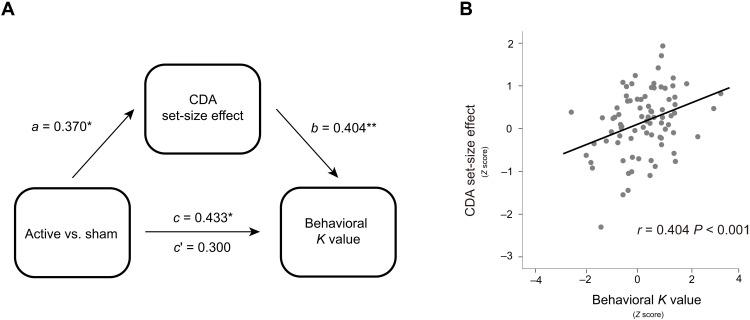
The CDA set-size effect mediated behavioral *K* values by 1064-nm tPBM. (**A**) Mediation model demonstrating the effect of 1064-nm tPBM on improved *K* values via increases in the CDA set-size effect. *a*, *b*, and *c* denote standardized beta coefficients of the direct path strength. *c*′ denotes the beta coefficient of path strength after controlling for changes in the CDA set-size effect. (**B**) Scatterplots of the behavioral *K* values (*Z* score) and the CDA set-size effect (*Z* sore) across all participants (active and sham session) in experiment 1 and experiment 2. *P < 0.05 and **P < 0.01.

Given the above notable relationship between the increase in CDA set-size effect and the increase in *K* values after 1064-nm tPBM, relative to sham sessions, we performed a mediation analysis (see Materials and Methods) to examine whether the effect of tPBM on WM capacity (reflected by *K* values) was mediated by the CDA set-size effect. Therefore, we considered the tPBM sessions (active versus sham) as predictors, WM performance (*K* values) as the predicted variable, and CDA set-size effect as a mediator (see Fig. 3A). This mediation analysis revealed a significant indirect effect of the CDA set-size effect (indirect effect: 0.300, 95% confidence interval: −0.107 to 0.706, *P* = 0.146; direct effect: 0.245, 95% confidence interval: 0.021 to 0.844, *P* = 0.040). The mediation analysis demonstrated the indirect effect of 1064-nm tPBM on the behavioral *K* values through an increase in the amount of information maintained in visual WM, as reflected by increases in the CDA set-size effect. We further correlated the CDA set-size effect with the *K* values across all sessions in experiment 1 and experiment 2. The correlation analysis showed that the change in the CDA set-size effect was strongly correlated with the change in the behavioral *K* values (*r* = 0.404, *P* < 0.001, confidence interval: 0.154 to 0.654; Fig. 3B), highlighting a robust relationship between the CDA set-size effect and WM improvement. This result is consistent with previous research (*29*) that CDA is indicative of the number of maintained objects in visual WM.

### The wavelength specificity of tPBM on the enhancement of WM capacity

**Fig. 4.**
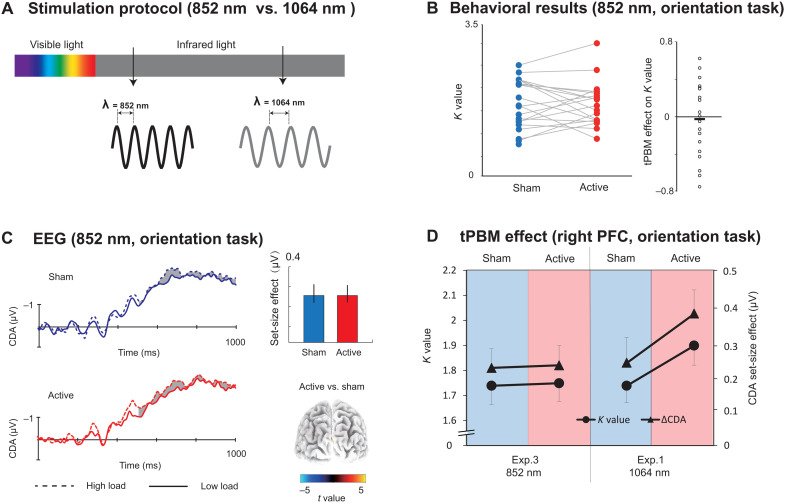
Results in experiment 3. (**A**) Stimulation protocol. Active tPBM was delivered by laser light with wavelength 852 nm over the right PFC in experiment 3 (black sine wave) or wavelength 1064 nm in experiments 1, 2, and 4 (gray sine wave). (**B**) The *K* values for tPBM stimulation (active and sham) for the orientation WM task in experiment 3. The circles indicate individual performance. (**C**) Grand average of ERPs for active 852-nm and sham tPBM sessions. The shading indicates the CDA set-size effect. Bar plots represent the average CDA set-size effect: blue, sham session; red, active session. Error bars represent SEM. 3D brain map (t-map) posterior view of the significant tPBM effect on CDA. (**D**) *K* values and CDA set-size effect for the orientation WM task in experiment 3 (852-nm tPBM) and experiment 1 (1064-nm tPBM).

In experiment 3, we investigated whether the changes in neural activity and behavior were specific to the wavelength of tPBM. An approximate wavelength of 850 nm has been used in near-infrared light for testing WM improvements due to its absorption maxima of CCO (*9*). This motivated us to test tPBM by using 852-nm laser light over the right PFC (Fig. 4A). We also sought to disambiguate the effects of tPBM and tissue heating. We hypothesized that if tPBM on the enhancement of WM capacity is not wavelength specific, but temperature dependent, then we should expect to find about the same enhancement in *K* values and CDA set-size effect with 852-nm tPBM and 1064-nm tPBM. We used the same power and stimulation duration for 1064-nm tPBM and 852-nm tPBM to ensure that they produced the same quantity of heat. First, we found that 852-nm tPBM did not change the participants’ behavioral *K* values for the orientation WM task compared with sham tPBM (*t*_18_ = 0. 381, *P* = 0.707, Cohen’s *d* = 0.085, two-tailed; Fig. 4B). The mean tPBM effect (active minus sham) on the *K* values for experiment 3 was −0.029 ± 0.088 (BF_10_ = 0.244 and Cohen’s *d* = 0.085). We compared the data between the 852-nm tPBM in experiment 3 and the 1064-nm tPBM in experiment 1 (Fig. 4D). A two-way mixed-effect ANOVA on *K* values further revealed a significant tPBM stimulation (active versus sham) and wavelength (1064 nm versus 852 nm) interaction (*F*_1,41_ = 4.474, *P* = 0.041, η*_P_*^2^ = 0.095), indicating that WM performance improved significantly only in active tPBM sessions applying 1064 nm but not an 852 nm. Similarly, 852-nm tPBM did not modulate the CDA set-size compared with sham tPBM for the orientation WM task (*t*_18_ = 0. 129, *P* = 0.899, Cohen’s *d* = 0.030, two-tailed; Fig. 4C). A two-way mixed-model ANOVA on CDA amplitudes further revealed a marginally significant tPBM stimulation (active versus sham) and wavelength (1064 nm versus 852 nm) interaction (*F*_1,41_ = 3.623, *P* = 0.064, η*_P_*^2^ = 0.080). These results suggest that the tPBM effect on WM is specific to the 1064-nm wavelength. We conclude that the behavioral and electrophysiological findings with 1064-nm tPBM are not explained by heating. This conclusion should be interpreted with caution due to the marginal significance observed in our electrophysiological results. Subjects also guessed at the chance level (hit rate = 47.8%), suggesting that they had no awareness of the 852-nm tPBM over the right PFC.

### 1064-nm tPBM on the left PFC did not enhance WM capacity

**Fig. 5.**
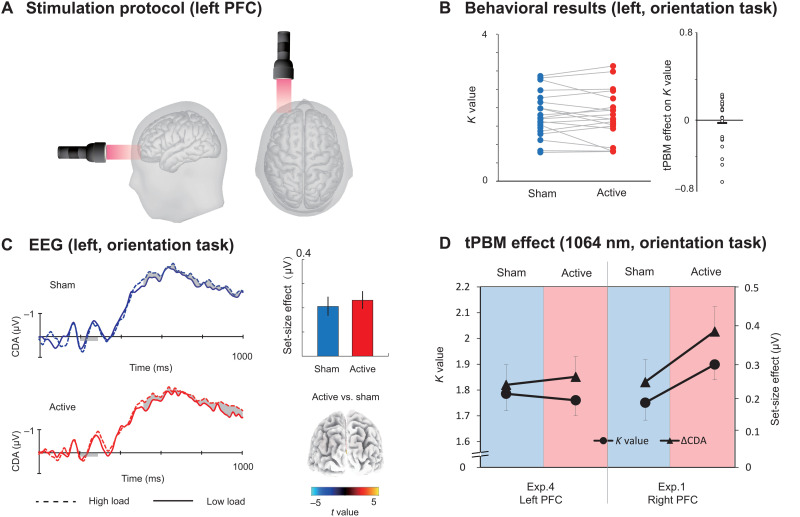
Results in experiment 4. (**A**) Stimulation protocol of experiment 4. Active tPBM was delivered by the laser with wavelength 1064 nm at the left PFC for a total of 12 min. (**B**) *K* values for tPBM stimulation (active and sham) were applied on the left PFC in experiment 4. Each circle indicates individual performance. (**C**) Grand average of ERPs for the 1064-nm and sham tPBM sessions in experiment 4. Shading indicates the CDA set-size effect. Bar plots represent the average CDA set-size effect: blue, sham session; red, active session. Error bars represent SEM, 3D brain map (t-map) posterior view of a significant tPBM effect on CDA. (**D**) *K* values and CDA set-size effect for the orientation WM task in experiment 4 (tPBM stimulation applied on the left PFC) and in experiment 1 (tPBM stimulation applied on the right PFC).

In experiment 4, we asked whether 1064-nm tPBM applied to the left PFC could enhance WM capacity (Fig. 5A). The task in experiment 4 is the same orientation WM task as experiment 1. Figure 4 shows that compared with sham tPBM, 1064-nm tPBM on the left PFC did not enhance the *K* values (*t*_18_ = 0. 381, *P* = 0.707, Cohen’s *d* = 0.085, two-tailed; Fig. 5B) nor the corresponding CDA set-size effect (*t*_18_ = 0. 129, *P* = 0.899, Cohen’s *d* = 0.030, two-tailed; Fig. 5C). The mean tPBM effect (active minus sham) on the *K* values for experiment 4 was −0.032 ± 0.058 (BF_10_ = 0.258 and Cohen’s *d* = 0.030). We compared the data between tPBM applied on the left PFC in experiment 4 and tPBM applied on the right PFC in experiment 1 (Fig. 5D). A two-way mixed-model ANOVA revealed significant interactions between tPBM stimulation (active versus sham) and location (left versus right) on both *K* values (*F*_1,41_ = 4.474, *P* = 0.041, η*_P_*^2^ = 0.095) and on the CDA set-size effect (*F*_1,41_ = 2.623, *P* = 0.044, η*_P_*^2^ = 0.098). These findings demonstrate that WM performance improved only when 1064-nm tPBM was applied to the right but not to the left PFC. Subjects also guessed at the chance level (hit rate = 46.9%) whether they received sham versus tPBM, suggesting that they had no awareness of the tPBM over the left PFC.

### Time course of the enhancement in WM after tPBM

**Fig. 6.**
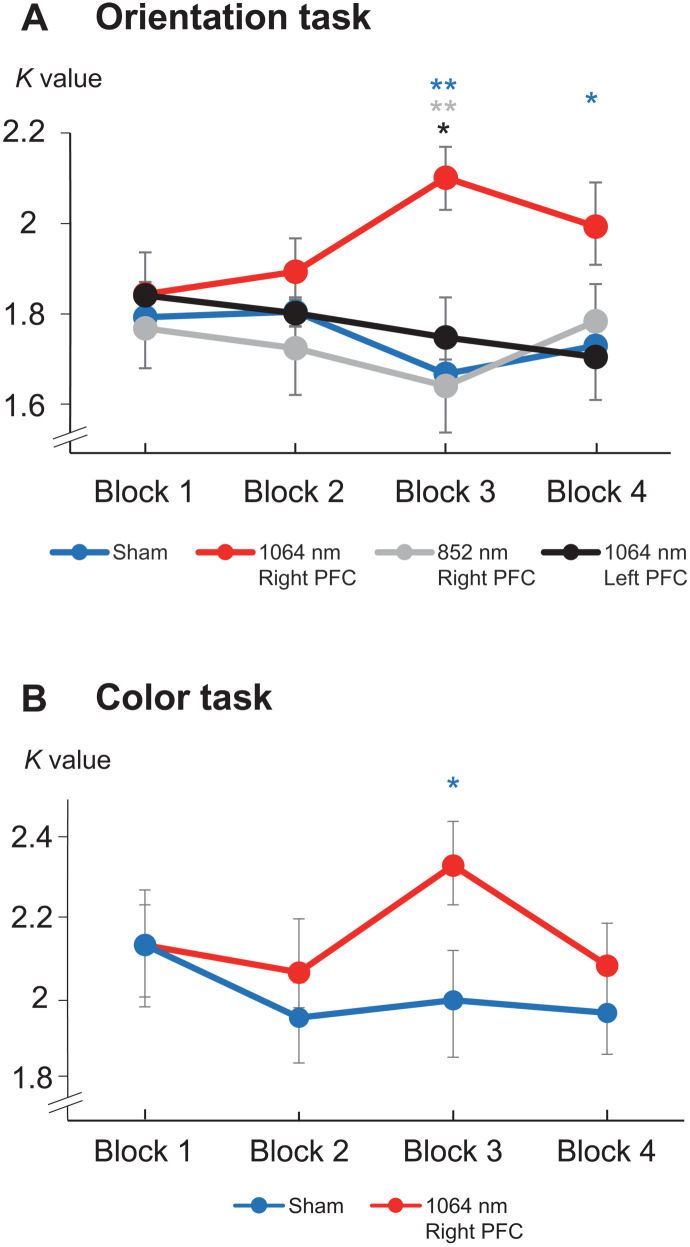
Time course of the behavioral results. (**A**) *K* values across the four blocks in the orientation WM task in experiments 1, 3, and 4. The blue line represents the sham session in experiment 1. (**B**) *K* values across the four blocks in the color WM task in experiment 2. The blue line represents the sham session in experiment 2. *P < 0.05 and **P < 0.01.

To investigate the behavioral enhancement across blocks, we calculated the *K* values of high-load conditions across the four blocks in all four experiments (Fig. 6). The paired *t* test showed that, relative to sham sessions, significant behavioral enhancements were found only during the late period in experiment 1 (block 3: *t_22_* = 3.840, *P* < 0.001, Cohen’s *d* = 0.768, two-tailed; block 4: *t*_22_ = 2.155, *P* = 0.041, Cohen’s *d* = 0.504, two-tailed) and experiment 2 (block 3: *t*_17_ = 2.137, *P* = 0.047, Cohen’s *d* = 0.431, two-tailed) with 1064-nm tPBM on the right PFC but not in other blocks or experiments (*P*s > 0.250). We further compared the *K* values in block 3 across experiment 1 and experiment 3 when applying tPBM over the right PFC, 1064-nm stimulation relative to 852 nm enhanced the *K* values (*t*_40_ = 2.795, *P* = 0.008, Cohen’s *d* = 0.838, two-tailed). When applying tPBM at 1064-nm wavelength over the right PFC relative to the left PFC, the *K* values were also enhanced (*t*_40_ = 1.959, *P* = 0.056, Cohen’s *d* = 0.580, two-tailed). No significant differences were found in other blocks (*P*s > 0.351).

### No evidence for event-related oscillatory activity after tPBM

We further analyzed the effects of tPBM on event-related desynchronization (ERD) or event-related synchronization (ERS) during the retention of WM. As shown in fig. S1, we found that active tPBM might amplify the set-size effect of theta ERS in experiment 1 (*t*_22_ = 2.487, *P* = 0.048, Cohen’s *d* = 0.403, two-tailed). This effect was not found in experiments 2, 3, and 4 (*P*s > 0.256). We further performed a correlation analysis to determine whether the set-size effect of prefrontal theta power was correlated with behavioral benefits on WM capacity in experiment 1. No significant correlation was found between theta ERS and changed in *K* values (*r* = 0.232 and *P* = 0.351). As shown in figs. S2 and S3, no significant differences in the set-size effect of alpha or beta ERD between the two tPBM sessions were found in each experiment (*P*s > 0.188). No significant relationships were found between the changes in the set-size effect of alpha (or beta) ERD and behavioral benefits (*P*s > 0.150). The ERD or ERS modulations were also not linked to the changes in the CDA set-size effect with stimulation. The reason might be as follows: (i) although both oscillatory activities and CDA are related to WM capacity (*27*, *30*), some studies further identified that they reflect dissociable processes of WM (*31*, *32*); (ii) poor statistical power for the oscillatory analysis explained by a lower signal-to-noise ratio. Recent findings proposed that cross-frequency coupling (e.g., phase-amplitude coupling) and long-range connections between distant cortical areas (e.g., phase locking value) might be related to network mechanisms supporting WM maintenance (*2*, *3*). Further work is needed to examine the tPBM effect on network changes during WM.

## DISCUSSION

Across four complementary EEG experiments, we provided the converging evidence that 1064-nm tPBM applied to the right PFC could improve visual WM capacity. In the first two experiments (experiments 1 and 2), the *K* values were enhanced for WM for orientation and color when 1064-nm tPBM was applied to the right PFC. Crucially, we found that the WM memory improvements were tracked by individual changes in CDA set-size effects. A mediation analysis revealed that the behavioral enhancements with tPBM were mediated by CDA set-size effects. Further studies demonstrated that effects on the capacity enhancement of visual WM were absent for tPBM applied at 852-nm tPBM (experiment 3) and to the left PFC (experiment 4).

### Improvement of CDA-identified WM capacity by 1064-nm tPBM on the right PFC

There has been recent excitement about tPBM as a safe, noninvasive, and simple modality for neuromodulation. In particular, it seems to be a promising tool for enhancing WM capacity in humans because several studies have shown notable improvements in tPBM-treated versus control groups as measured by behavioral responses in WM tasks (*22*, *23*). Our results from experiments 1 and 2 do not only complement those findings, but also the design and outcome of these two experiments add novel insight.

First, WM involves multiple cognitive processes, including perceptual encoding, selective attention, and motor execution. More specific measures of WM capacity (e.g., *K* values and CDA set-size effect) bring us closer to identifying specific enhancements by tPBM. Thus, we designed an EEG study to investigate the post-tPBM effects on the CDA reflecting the individual visual WM capacity. Because the CDA set-size effect reflects the number of objects online-held in visual WM (*29*), our ERP results help to establish that 1064-nm tPBM on the right PFC boosts visual WM capacity. It also corroborates and extends existing findings that active maintenance of visual information in the occipitoparietal cortex could be boosted by enhancing the contribution of the right PFC in visual WM maintenance (*33*). We further established neurophysiological links between 1064-nm tPBM and subsequent WM capacity, by demonstrating that the CDA during the retention served as a mediator. Our findings suggest that increased WM from 1064-nm tPBM might stem from the right PFC stronger engaging parietal areas as reflected by the increased CDA set-size effect. However, given that both hemodynamic effects (*34*) and EEG activity (*35*) from the IPS are correlated with WM capacity, we could not pinpoint whether the CDA set-size effect plays a causal role in enhancing WM capacity or whether it is a byproduct of hemodynamic activity. Given that the frontoparietal network (FPN), including the supplementary motor area, PFC, and IPS are thought to be important for WM (*36*), we inferred that the 1064-nm tPBM might increase metabolism (e.g., providing more ATP) in the right PFC with positive benefits for the FPN network.

Second, EEG signals in response to tPBM stimulation have been reported recently from resting brain data, and they were analyzed to identify frequency-specific changes in EEG power (*16*, *37*, *38*). Our study demonstrates that WM-specific ERPs also are modulated by tPBM stimulation. This contribution is important for researchers working in the field of tPBM who wish to understand the underlying electrophysiological effects of tPBM.

### Possible changes in the brain network by 1064-nm tPBM in the right PFC

The LORETA results showed an effect of ERPs from the IPS when the stimulation was applied over the right PFC. There are several explanations for this observation. A recent study (*38*) reported that 1064-nm tPBM on the right PFC enables notable increases in alpha power in several brain networks at rest (i.e., the default mode network, executive control network, frontal-parietal network, and lateral visual network). Another independent set of studies from the same group revealed that 1064-nm tPBM on the right PFC increased hemodynamic activities across the entire cortical region and enhanced topographical functional connectivity between the right PFC stimulation site and parietal regions (*5*, *39*). Together, these studies show that 1064-nm tPBM on the right PFC of the resting human brain modulates both regional-specific activity and functional connectivity as observed in EEG and hemodynamic data. While these results were derived from the intervention administered during the resting state, it is reasonable to expect that tPBM would also alter the task-related activity between the right PFC and the parietal regions as we here report. We posit that the CDA set-size effect that we report is related to connectivity changes between the right PFC stimulated by tPBM and the IPS generating the CDA. In support, it has been suggested that external interventions can result in increased functional connectivity between the PFC and IPS during WM operations (*3*, *40*). The tPBM applied in this study is likely to result in changes and enhancement in metabolism and hemodynamics (*41*, *42*). It has been suggested that the neurovascular coupling between hemodynamic activity and EEG is suggested to play a role in visual information processing (*43*, *44*). Thus, further EEG-functional near-infrared spectroscopy (fNIRS)/functional magnetic resonance imaging (fMRI) studies would help to gain a better understanding of the underlying mechanism of the beneficial effects resulting from tPBM.

### Wavelength-dependent effect of right PFC tPBM on visual WM capacity

Wavelength is a major illumination parameter of tPBM within an “optical window” in the red–to–near-infrared optical region, as it greatly determines the photon absorption of molecular target CCO (*10*). Literature reviews of tPBM have exhibited a wide range of wavelengths applied in both animals and humans (*8*–*11*). The most common wavelengths used are in the range of 600 to 900 nm, particularly at 660, 810, and 850 nm (*45*). The reason applying those wavelengths in PBM or tPBM is because CCO has light absorption peaks at 660 and 800 to 850 nm (*46*). Thus, these specific wavelengths might promote metabolic effects. On the other hand, 1064-nm laser stimulation has demonstrated effects on the enhancement of human cognition (*22*, *23*), as also reported here. However, it is unclear whether tPBM by other wavelengths from the laser would create similar effects on CCO activity and vascular hemodynamics. Such insight would be exceptionally helpful to researchers, clinicians, and potential manufacturers in the PBM field. A better understanding of the wavelength-specific tPBM effects on mitochondrial and hemodynamic activities in the human brain would facilitate the optimal selection of wavelengths to optimize the outcomes. Specifically, it is also crucial to further uncover if tPBM-induced behavioral and CDA enhancements in visual WM capacity are wavelength dependent.

Accordingly, we designed experiment 3 by switching only the laser wavelength from 1064 to 852 nm and thus to investigate wavelength-specific effect in WM. The ideal laser light of tPBM should have the theoretical advantage of traveling deeper into the tissues of the human body and the best absorption of light by CCO. In reality, there is a trade-off between the absorption of light by CCO and the depth of penetration. In the absorption spectra, the photon absorption peak of CCO is close to 852 nm. However, light at this wavelength is more scattered preventing light from travelling deeply through tissue. In comparison, the longer 1064-nm wavelength allows for deeper tissue penetration but less absorption of light by CCO. Here, we used tPBM with 1064 nm (good penetration) and 852 nm (good absorption) to find the optimal wavelength for tPBM. We found that tPBM with 1064 nm specifically boosted the behavioral performance of the participants and the CDA set-size effect in the WM task. Such behavioral and EEG modulations were not observed for tPBM at 852 nm. These results suggest that 1064 nm is a better wavelength for tPBM with photon delivery into the PFC due to its reduced tissue scattering (*42*). Note that tPBM with 852 nm had the same laser energy and the same stimulation duration as tPBM with 1064 nm and thus resulted in comparative putative heating. It can therefore also be considered as an active-controlled group to eliminate the exogenous thermal effect that would bias or confound the observed changes. To our knowledge, these results provide the first evidence of wavelength-specific WM capacity improvement by tPBM. Meanwhile, uncertainty remains about the photobiomodulation physiological mechanism with respect to the choice of wavelength. Further research is needed to determine how variation in illumination parameters, such as power density, wavelength, treatment timing, and pulse structure, would affect the memory-enhancing effects of tPBM.

### Site-dependent effect of 1064-nm tPBM on visual WM capacity

To examine site-dependent effect, we designed experiment 4 by switching the stimulation site from the right PFC to the left PFC while keeping the rest intervention parameters the same. The results showed that the contribution of tPBM to improving WM capacity is specific to the right PFC. It supports the notion that the right PFC is more closely associated with information maintenance in visuo-spatial WM (*26*). Given previous work showing that WM capacity could be modulated by increasing the PFC excitability (*47*), we here offer a new effective intervention to enhance visual WM capacity and provide new evidence for a causal role of right PFC for WM maintenance. In the past decade, other NIBS technologies such as tDCS have been shown to enhance WM performance by increasing cortical excitability. Our null effect of left PFC stimulation with tPBM in experiment 4 is consistent with the observation that applying anodal tDCS over the left PFC does not improve visual WM capacity (*48*). However, other studies have shown that applying anodal tDCS over the left PFC could improve the behavioral performance of verbal WM (*49*–*51*). Although both the left and right PFC might have general beneficial effects from NIBS technology, as the “central executive” is an important unit in the classic storage-and-processing mode of WM maintenance (*52*), a recent review noted out the distinct neural mechanisms between visual WM and verbal WM (*53*).

### Benefits of tPBM and potential applications

Our findings also showed differences between tPBM and other NIBS technologies. Unlike the WM capacity–dependent changes after tDCS (*28*, *54*), our results showed WM improvements in participants with both low and high WM capacity after 1064-nm tPBM. We suggest that tPBM is a useful tool for improving the upper limits of WM by augmenting the neural metabolism of the PFC. Recent studies have attempted to improve WM performance by modulating brain activity through within-trial rhythmic entrainment using alternating transcranial current stimulation (*2*) and repetitive magnetic stimulation (*3*). These studies showed that these NIBS technologies can bring the peak and online benefit of behavior gains by modulating temporally neuronal activities. However, participants subjected to 1064-nm tPBM performed better after the first two blocks than those who received the sham, 852-nm, or left tPBM. It seems that tPBM produces behavioral improvements after several minutes of stimulation but not immediately. These observations might stem from tPBM, which required the involvement of multiprocess of brain activity, unlike that tDCS induces the change of the underlying cortex by causing the neuron’s resting membrane potentials to depolarize or hyperpolarize. Consistently, previous research on tPBM yielded an optimal impact on tasks when administered over several minutes (*55*). Note that this effect decreased in block 4, and it seems that 1064-nm tPBM has limited sustained effects. The limitations of the present studies include the relatively short follow-up period. Further study should be conducted to explore the time course of the behavioral effects of the stimulation. To our knowledge, the reported literature demonstrates that behavioral benefits of tPBM almost all applied stimulation to sites on the forehead. Because of the absorption of light by the hair, other areas such as the IPS may receive limited effects if being stimulated directly by tPBM (*45*). Further work should break the barrier between hair and laser light to expand the applicability of tPBM to the whole brain.

In conclusion, our study provides novel and compelling evidence that 1064-nm tPBM applied to the right PFC enhances visual WM capacity in humans. Considering that several disorders, such as attention-deficit hyperactivity disorder (ADHD), schizophrenia, and AD, show a decline in WM capacity (*56*–*58*), our observations offers a safe, effective, cost-effective, and noninvasive brain intervention tool for clinical applications. To date, there are no side effects or harm associated with tPBM reported in the literature. In addition, compared with other NIBS technologies that may produce a certain tingling sensation or acoustic noise, tPBM is silent. Thus, tPBM is suitable for promoting clinical applications in individuals with memory dysfunction, such as patients with ADHD and AD. However, tPBM will depend on stimulation parameters, such as the power density, wavelength, dosage, and location. Further research work is needed from biophysical and neurobiological aspects to use the full potential of tPBM before it can be applied to improve cognition in healthy and clinical populations.

## MATERIALS AND METHODS

### Participants

Neurologically normal college students (*n* = 90) with normal or corrected-to-normal vision participated in four experiments. Of these, 27 participated in experiment 1 (five males, mean age = 22). No statistical methods were used to predetermine the sample size, but the sample size was chosen to be adequate to obtain robust results as determined by preliminary experiments. Because the identified tPBM effect in experiment 1 was robust, we set the sample size to 21 in experiments 2 to 4 (experiment 2: seven males, age range = 22.8 ± 3.8; experiment 3: eight males, age range = 22.7 ± 4.1; experiment 4: seven males, age range = 22.8 ± 4.0). Data from 11 participants (four in experiment 1, three in experiment 2, two in experiment 3, and two in experiment 4) were excluded because of incomplete data or low EEG quality. The Institutional Review Board approved the experimental procedures of Beijing Normal University, and informed consent was obtained from each participant.

### Experimental protocol

Each participant only participated in one of four experiments. Each experiment consisted of one active tPBM session and one sham tPBM session completed on the first and the seventh day. The order of the sessions was counterbalanced across participants (see Fig. 1B). On the eighth day, participants were required to report (or guess) which session was the active tPBM session. Before EEG recordings, all subjects participated in a training block to ensure that they could perform the tasks above the chance level, and we checked for potential EEG artifacts.

### tPBM protocol

Four experiments were administered using a diode-pumped solid-state laser with a linewidth of ±1 nm (Model JL-LS-100 developed by Jieliang Medical Device Inc., Jiangxi, China). The measured uniform laser beam has an area of 13.57 cm^2^ (4 cm in diameter) and a continuous power output of 2271 mW, resulting in an irradiance or power density of 167 mW/cm^2^ (2271 mW/13.57 cm^2^ = 167 mW/cm^2^). At this power level, the energy emitted by the laser is one-fifth of the skin’s maximum permissible exposure (the exposure not deemed harmful to tissue and causing no detectable physical damage or imperceptible heat). The stimulation was handheld. The stimulation site in our experiment was centered on the FP2 electrodes (experiments 1, 2, and 3) or the FP1 electrode (experiment 4) based on the 10-20 system used for EEG electrode placement (Fig. 1A, top). Each subject was instructed to sit on a chair, which was adjusted to ensure comfort throughout the measurement. The ambient lighting of the room was eliminated to ensure that it did not contaminate the laser light. Participants were instructed to wear protective eyewear and keep their eyes closed, as required by the laser manufacturer and the Beijing Normal University Laser Safety Program. In the active tPBM session, the area stimulated (a 60 s/cycle, total laser energy per cycle = 2.271 W × 60 s = 136.26 J/cycle) alternated between sites medial and lateral to the FP2 or FP1 for 12 min before EEG recording. The sham tPBM session received two brief 0.5-min stimulations (at the beginning and end of the 12-min period) to the intended site on the forehead, separated by 11 min of no stimulation (the laser power was tuned down to 0 W; Fig. 1A, bottom). Thus, the sham tPBM session received approximately 1/12th of the cumulative energy density compared to the active session. This 0.5-min treatment was a necessary part of the active placebo session by providing a similar subjective experience to the active tPBM session without producing physiological or cognitive effects (*22*, *59*). For tPBM stimulation, 1064 nm was used for experiments 1, 2, and 4; 852 nm was used for experiment 3. The two wavelengths were controlled to release equal optical energy. Each session lasted 45 to 60 min (12-min tPBM stimulation, 8-min rest, and 25- to 30-min EEG recording). The active and sham sessions were separated by at least 1 week to avoid overlapping effects.

### WM task

The stimuli were presented on a 48 × 27 cm^2^ liquid crystal display monitor (1200 × 768 pixels, 120-Hz refresh rate) with a homogeneous light gray background (12 cd/m^2^; Red Green Blue (RGB): 125, 125, and 125) at a distance of 65 cm. At the beginning of each trial, a 200-ms central arrow cue instructed the participants to remember the left or the right hemifield objects. Next, the memory array was presented for 100 ms, followed by a 900-ms interval. Then, the probe array was presented for a maximum of 2000 ms or until response. Participants were instructed to respond as quickly and accurately as possible whether the orientation or color of the objects in the cued hemifield had changed after the WM delay.

In the orientation WM task, all memory arrays were presented within two 4° × 7.3° rectangular regions that were centered 3° to the left and right of a black central fixation cross (0.5 cd/m^2^, 0.4° × 0.4°). Each memory array consisted of two or four red bars (2° in length and 0.5° in width) in each hemifield selected with a random orientation between 0° and 180°, with the constraint that the orientations among bars within a hemifield differed by at least 20°. The bars’ positions were randomized in each trial, with the constraint that the distance between the bars within a hemifield was at least 2° (center to center). The orientation of one bar in the probe array was different from the corresponding object in the memory array in 50% of the trials in each hemifield; the orientations of two arrays were identical in the remaining trials. In the color WM task, each memory array consisted of two or four colored squares (1° × 1°) in each hemifield. Each square was selected randomly from a set of nine colors (red, green, blue, yellow, violet, pink, orange, black, and white). In the low-load condition, one square was presented in each quadrant. In the high-load condition, two squares were presented in each quadrant. In the probe array, the color of one square was different from the corresponding object in the memory array in 50% of the trials in each hemifield; the two arrays’ colors were identical in the remaining trials. Each session involved eight blocks (four low-load blocks and four high-load blocks, randomized across blocks). Each block contained 60 trials, and an approximately ~1-min break separated adjacent blocks. In total, we collected 960 trials within ~30 min in each experiment per participant. Experiments 3 and 4 were identical to the same orientation WM task in experiment 1, except that the participants were assigned to active tPBM with different wavelengths (852 nm) in experiment 3 and with another site (left PFC) in experiment 4.

### EEG recording and analysis

The EEG signals were recorded while the participants performed the tasks. The EEG data were acquired using a SynAmps EEG amplifier and the Curry 8.0 package (NeuroScan Inc.) from a Quick-cap with 64 silver chloride electrodes arranged according to the international 10-20 system. To detect eye movements and blinks, vertical eye movements were recorded from two vertical electrooculogram electrodes placed 1 cm above and below the left eye, while horizontal eye movements were recorded from two horizontal electrooculogram electrodes placed at the outer canthus of each eye. All electrodes, except those for monitoring eye movements, were referenced online to the left mastoid. Electrode impedance was kept below 5 kilohm. The EEG was amplified at 0.01 to 200 Hz and digitized online at a sampling rate of 500 Hz.

The data were processed in MATLAB (MathWorks Inc., Natick, MA) using the ERPLAB toolbox and custom codes. They were preprocessed by applying a 0.1- to 40-Hz bandpass filter and rereferencing data offline to the average of all electrodes. The EEG data were then segmented relative to memory array onset (from −200 to 1000 ms). Independent component analysis was performed to correct eye-blink artifacts by semiautomatic routines for the segmented data. Epochs were automatically excluded if the EEG exceeded ±100 μV at any electrode or if the horizontal Electro-Oculogram (EOG) exceeded ±30 μV from 0 to 500 ms around cue array onset. Epochs that continued to show artifacts after this process were subsequently detected and removed by the eye. Data from nine participants (three in experiment 1, two in experiment 2, two in experiment 3, and two in experiment 4) were discarded because of the high ratio of excluded trials (>40% of trials). Among the participants’ final set, an average of 12.3% of trials per participant (range, 0.4 to 27.6%) were rejected because of artifacts.

We focused on the ERP triggered by the memory array. The baseline correction was calculated for 200 ms before memory display onset in each trial. The trials were then averaged for each condition to create the ERP response. Contralateral waveforms were computed by averaging the right electrode sites for trials on which to-be-remembered objects occurred on the left side with the left electrode sites for trials on which to-be-remembered objects occurred on the right side. Ipsilateral waveforms were computed by averaging the right electrode sites for trials on which to-be-remembered objects occurred on the right side with the left electrode sites for trials on which to-be-remembered objects occurred on the left side.

The CDA was measured at the posterior parietal sites (PO7/PO8) as the difference in mean amplitude between the ipsilateral and contralateral waveforms, with a measurement window of 500 to 1000 ms after the onset of the cue array. In this study, the memory display could induce an N2pc before the CDA component (*32*). To obtain a pure CDA measure without contamination of the N2pc component, we began the CDA measurement period at 500 ms, by which time the N2pc had ordinarily terminated. Note that the contralateral waveform of the target was the average of the left-hemisphere electrodes when the target was in the right visual field and the right-hemisphere electrodes when the target was in the left visual field. Similarly, the ipsilateral waveform for the target was the average of the left-hemisphere electrodes when the target was in the left visual field and the right-hemisphere electrodes when the target was in the right visual field.

During ERP analysis in the visual WM task, we detected the difference in CDA between groups in active and sham sessions with *t* statistics analysis. For each comparison, a test was calculated for time samples in ERP components with 5000 random permutations.

For the ERS or ERD analysis, artifact-free trials were decomposed using a Morlet wavelet–based analysis from 4 to 30 Hz in 1-Hz steps implemented in the related package Fieldtrip in the MATLAB environment. We subtracted the trial-average activity in the time domain from the EEG activity of every single trial to avoid the time frequency of power being disturbed by the ERP in oscillatory signals. Load-dependent power changes in theta (4 to 7 Hz), alpha (8 to 12 Hz), and beta (14 to 30 Hz) during retention were calculated to examine the WM-related oscillatory activity between sham and active tPBM sessions. Changes with respect to a baseline over time were referred to as ERS or ERD. The percentage of ERS or ERD was calculated by following the procedure reported elsewhere (*30*): (i) the mean baseline (−400 to 0 ms before memory display onset) amplitude was subtracted from the power estimates at each time point, and (ii) the difference was divided by the mean baseline. Our electrodes of interest were occipitoparietal electrodes (P3/4, PO3/4, O1/2, PO7/8, P7/8, and POz) and middle frontal electrodes (FP1/2, AF1/2, F3/4, F1/2, and F_Z_) based on previous findings (*30*, *60*). The set-size effects were defined as the change in amplitudes from set-size two to set-size four conditions.

### Source locations

The three-dimensional (3D) distribution of the tPBM effect (active minus sham) in electrical activity was analyzed for each subject using LORETA software (*61*). Localization of the CDA set-size effect between the active and the sham session was assessed by voxel-by-voxel paired *t* tests of the LORETA estimates. To correct for multiple comparisons, a nonparametric permutation test was applied (*P* < 0.050, determined by 5000 randomizations). Last, the significant result values were projected on a 3D brain model.

### Statistical analysis

Mediation analysis for multilevel data was performed in the SPSS statistics package (version 20.0). Two models were built in the analysis: (i) a linear model to test the relationship between the tPBM session and the behavioral *K* values and (ii) a generalized linear model established with “active versus sham” as the predictors, “CDA set-size effect” as the mediator, and “behavioral *K* value” as the predicted variable. The direct and indirect effects were then obtained by contrasting these two models. The null hypothesis was tested by examining whether zero was within the 95% bootstrap confidence intervals.

We conducted a Bayesian analysis (conducted with JASP software v.0.13.1.0) to test the null hypothesis. Bayes factor analyses with default priors (*r* = 0.707) were performed on the EEG data (BF_10_ > 1: support for H_1_ over H_0_; BF_10_ < 0.333: substantial evidence for the null hypothesis).

## Notes

View/request a protocol for this paper from *Bio-protocol*.
