# Supplementary material for: Transcranial photobiomodulation enhances visual working memory capacity in humans
Source: Sci Adv. 2022 Dec 2;8(48):eabq3211. doi: 10.1126/sciadv.abq3211 (PMC10936045; doi:10.1126/sciadv.abq3211)
Supplement: Supplementary file 1 — Figs. S1 to S3 [file sciadv.abq3211_sm.pdf]

Science Advances
AAAS

Supplementary Materials for

Transcranial photobiomodulation enhances visual working memory capacity in humans

Chenguang Zhao *et al.*

Corresponding author: Yan Song, songyan@bnu.edu.cn

Sci. Adv. **8**, eabq3211 (2022)
DOI: 10.1126/sciadv.abq3211

This PDF file includes:

Figs. S1 to S3

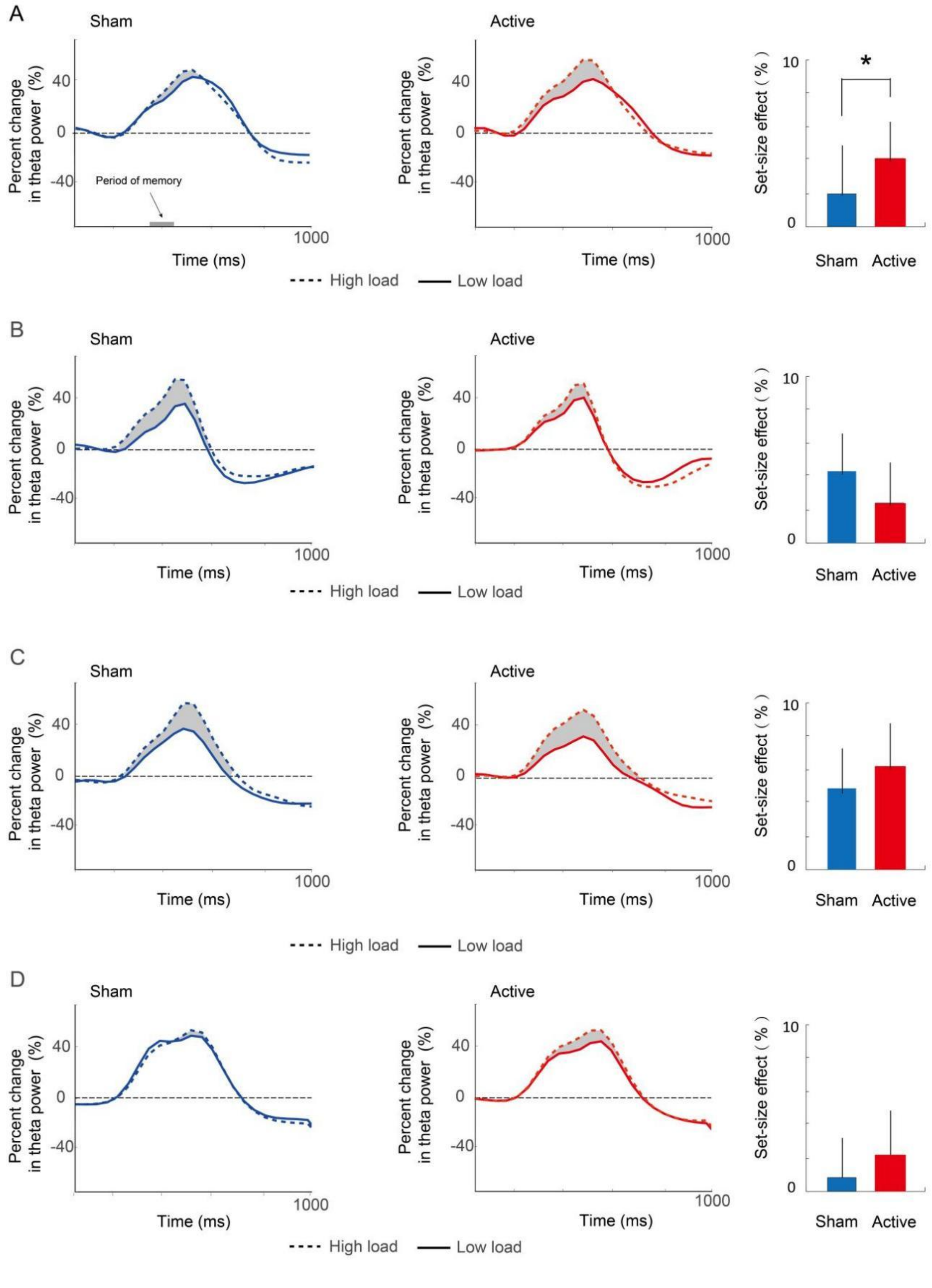

Figure S1. Theta band ERS results for all experiments. The theta band ERS for two sessions (active-tPBM and sham-tPBM) in Experiment 1 (**A**), Experiment 2 (**B**), Experiment 3 (**C**), and Experiment 4 (**D**). Averaged ERS in theta power was observed at the middle frontal electrodes after the onset of the memory array indicated by the grey box. Shading indicates the set-size effect of theta ERS. Right panel, mean set-size effect of theta power enhancement during the retention interval. Error bars represent SEM. * $p < 0.05$

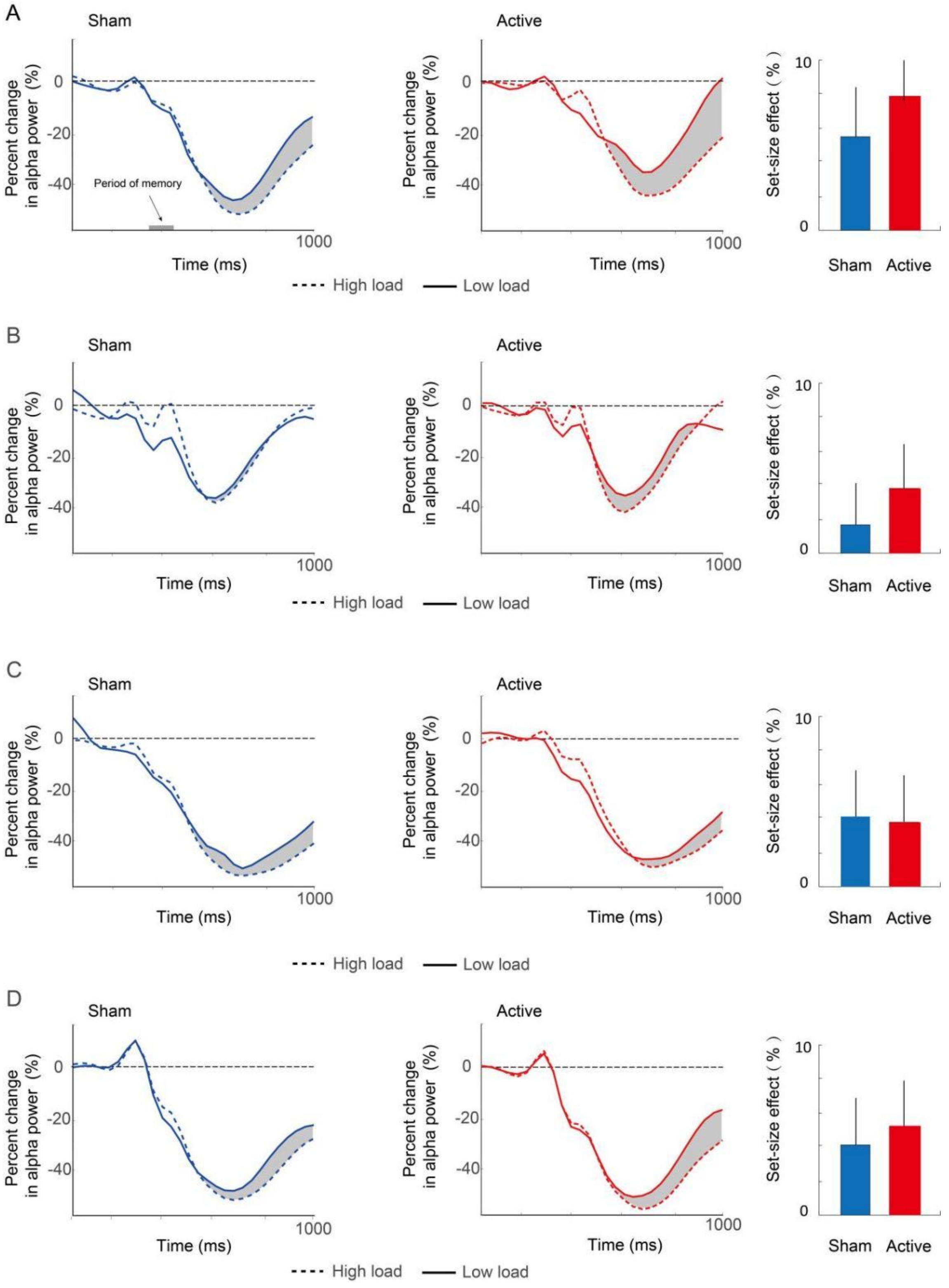

Fig. S2. Alpha band ERD results for all experiments. Alpha band ERD for two sessions (active-tPBM, sham-tPBM) in Experiment 1 (**A**), Experiment 2 (**B**), Experiment 3 (**C**), and Experiment 4 (**D**). Averaged ERD in alpha power was observed at the parieto-occipital electrodes after the onset of the memory array, as indicated by the grey box. Shading indicates the set-size effect of alpha ERD. Right panel, mean set-size effect of alpha band ERD during the retention interval. Error bars represent SEM. No significant difference in the set-size effect of alpha band ERD between active and sham tPBM was found in each experiment ($ps > 0.150$).

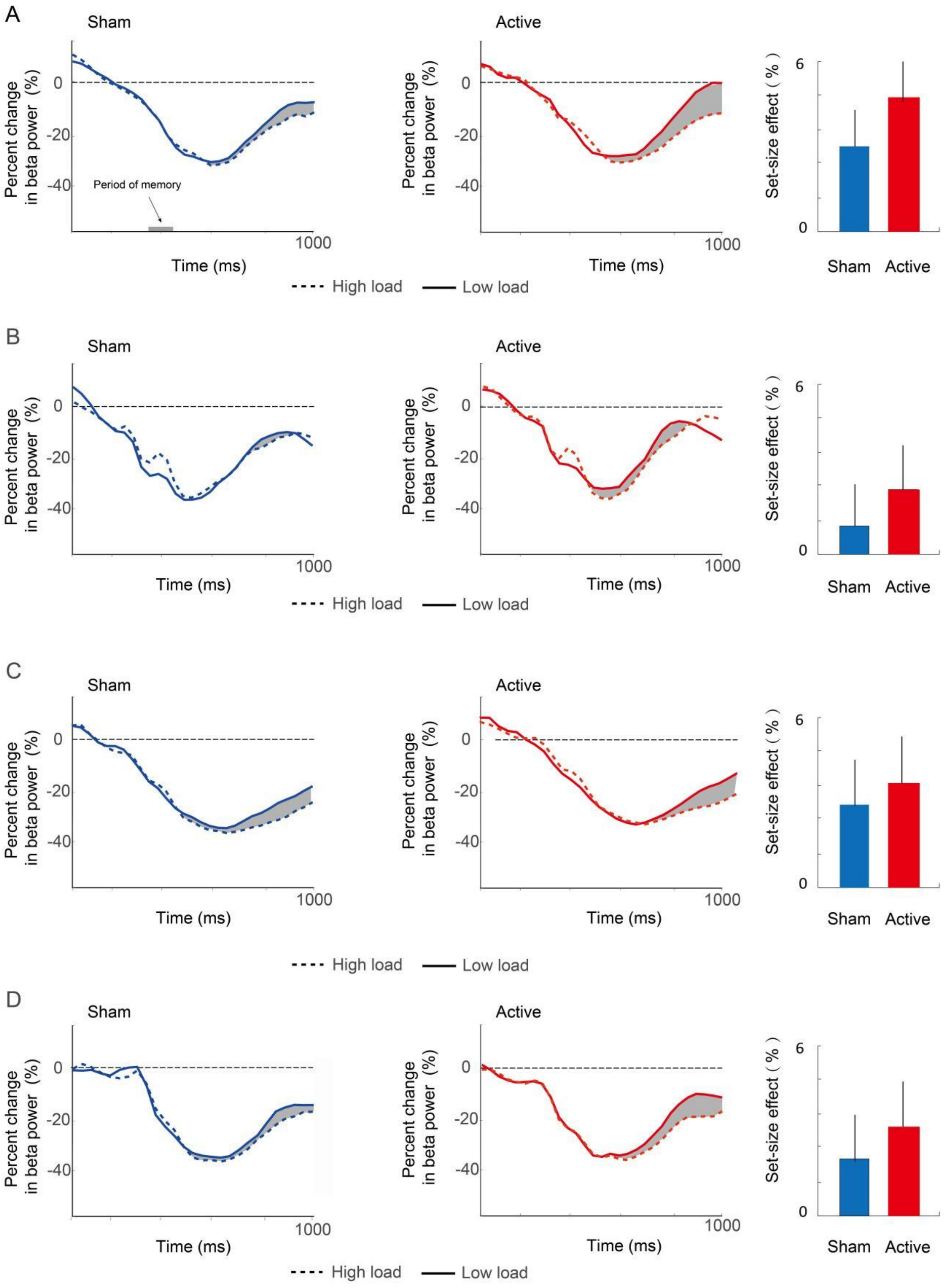

Figure S3. Beta band ERD results for all experiments. The beta band ERD for two sessions (active-tPBM and sham-tPBM) in Experiment 1 (**A**), Experiment 2 (**B**), Experiment 3 (**C**), and Experiment 4 (**D**). Averaged ERD in beta power was observed at the middle frontal electrodes after the onset of the memory array indicated by the grey box. Shading indicates the set-size effect of beta ERD. Right panel, mean set-size effect of beta power suppression during the retention interval. Error bars represent SEM. No significant difference in the set-size effect of beta ERD between active and sham tPBM was found in each experiment ($ps > 0.227$).
